# Demographic, Chemical, and *Helicobacter pylori* Positivity Assessment in Different Types of Gallstones and the Bile in a Random Sample of Cholecystectomied Iranian Patients with Cholelithiasis

**DOI:** 10.1155/2021/3351352

**Published:** 2021-08-09

**Authors:** Mohammad Bagher Jahantab, Amir Abbas Safaripour, Sajad Hassanzadeh, Mohammad Javad Yavari Barhaghtalab

**Affiliations:** ^1^Department of General Surgery, Shahid Beheshti Hospital, Yasuj University of Medical Sciences, Yasuj, Iran; ^2^Department of Internal Medicine, Imam Sajjad Hospital, Yasuj University of Medical Sciences, Yasuj, Iran

## Abstract

**Background:**

The occurrence of stones in the gallbladder or common bile duct and the symptoms and complications they cause is called gallstone disease. The symptoms of gallstone disease range from mild, nonspecific symptoms to a severe right quadrant abdominal pain. Characteristics of gallstone types in an Iranian population have not been well studied before and there are very limited studies on the demographic pattern of stone types in our country, so this study is one of the first studies on its kind on the epidemiology of gallstone types in Iran. As information on chemical components of the stone will help in the management and prevention of gallstones, in this study, we aimed to do chemical component analysis of gallstones including cholesterol, bilirubin, and calcium. Given the conflicting reports about the relationship between *H. pylori* infections and gallstone formation, this study aimed to investigate the relationship between *H. pylori* positivity in the bile specimen of Iranian patients with cholelithiasis and formation and type of stone.

**Methods:**

This prospective study reviewed a total of 196 patients who underwent cholecystectomy for symptomatic cholelithiasis at Shahid Beheshti Training and Research Hospital affiliated to the Yasuj University of Medical Sciences between September 2015 and May 2018. Chemical analysis of gallstone components performed using the colorimetry method. Microbiological analysis for *H. pylori* was done using the OnSite *H. pylori* Ag Rapid Test on the bile sample. For the validation test of bile, the *H. pylori* Rapid Stool Ag Test on stool was used, and Cohen's Kappa statistical analysis was done next.

**Results:**

There were significant associations between the stone types and age, chemical composition of the stones such as calcium, cholesterol, and bilirubin levels, and also *H. pylori* positivity and cholesterol and bilirubin levels; however, no significant association was found between the stone types and sex, *H. pylori* positivity and age, sex, stone types, and calcium level. The main bile and validity tests were matched to the substantial agreement according to Cohen's Kappa analysis. The most common drugs used were proton pump inhibitors, nonsteroidal anti-inflammatory drugs, antihypertensive drugs, and oral contraceptives.

**Conclusions:**

This study suggested that the chemical composition of the stones could predict the presence of bacteria, there is no correlation between H. pylori and gallstone formation, and some of the drugs could be predisposing factors for gallstones. This work provides an objective basis for further research into gallbladder stone formation; meanwhile, it has great significance in the treatment and prevention of gallbladder stones. Trial registration. The project was found to be in accordance to the ethical principles and the national norms and standards for conducting research in Iran with the approval ID IR.YUMS.REC.1399.147 and date 2020.09.23, and this project is the result of a residency dissertation to obtain the specialty in general surgery, which has been registered with the research project number 960159 in the Vice Chancellor for Research and Technology Development of the Yasuj University of Medical Sciences, Yasuj, Iran, URL: https://ethics.research.ac.ir/EthicsProposalViewEn.php?id=160634.

## 1. Background

The occurrence of stones in the gallbladder or common bile duct and the symptoms and complications they cause is called gallstone disease. Chronic cholecystitis due to gallbladder stone or cholelithiasis or symptomatic gallbladder is a prolonged mechanical or functional disorder of abnormal gallbladder emptying. The symptoms of gallstone disease range from mild, nonspecific symptoms to a severe right quadrant abdominal pain. Most of the patients have recurrent pain attacks (acute biliary colic), but when pain lasts more than 24 hours, it requires urgent surgical intervention [1, 2].

Several factors such as chemical composition, structure, morphology, and microbiological findings have been reported as different mechanisms leading to the formation of gallstones [3, 4]. Gallstones form as a result of a settling process in which solid particulates settle to the bottom of a liquid and form a sediment. The major organic solutes in the bile are bilirubin, bile salts, phospholipids, and cholesterol [5]. Gallstones are classified by their cholesterol content into 3 types as cholesterol stones (cholesterol content ≥70%), pigment stones (cholesterol content ≤30%), and mixed stones (30% ≤cholesterol content ≤70%) [6]. Nearly eighty percent of the gallstones are cholesterol gallstones, and 20% are pigment stones consisting of bilirubin and calcium, two components present in the bile. Calcium-bilirubin salts form outside surfaces with a high tendency of cholesterol to adhere to, and in addition, there is a nidus of calcium in almost all types of gallstones [7].

For the first time in history in 1966, Kaufman et al. suggested the role of bacterial infection in the pathogenesis of the pigmented gallstones [8]; afterward, several studies were established in this regard. The main elements in the formation of pure cholesterol gallstones are cholesterol saturation and solubility. As it takes a long time for the establishment of cholesterol gallstones, the entrenched bacteria would be destroyed and die, so no causative bacteria have been isolated to date [9]. Urease is the enzyme that plays an important role in the bacteria-induced stone formation. This enzyme hydrolyzes urea into ammonia and bicarbonate. Ammonia would cause an increase in pH, and this causes the generation and precipitation of an insoluble form of calcium. This mechanism, which was described above, is a common pathway among different types of stones [7]. Therefore, bacterial infection is a precipitating factor in the pathogenesis of cholesterol gallstones [9]. Another theory is that the bacterial products' accumulation in the bile would help nucleation of cholesterol, hydrolysis of conjugated bilirubin, and precipitation of insoluble organic calcium salts [10].

A few studies have reported infectious agents related to the pathogenesis of cholesterol gallstones. The polymerase chain reaction (PCR) was suggested by Swidsinski et al. for identification of the bacteria in cholesterol gallstones. Lee et al. found *Escherichia coli* (*E. coli*) and *Pseudomonas* as the pathogens in cholesterol gallstone formation [9]. *Helicobacter pylori* (*H. pylori*) is a spiral, microaerophilic, gram-negative bacterium responsible for this situation [4]. It lives in the stomach and is related to acute and chronic gastritis, gastric and duodenal ulcer, gastric and pancreatic adenocarcinoma, and lymphoma of gastric mucosa-related lymphoid tissue (MALToma) [3, 11, 12]. The presence of *H. pylori* outside the stomach and diseases associated to this situation have been investigated [3]; such as, in primary sclerosing cholangitis and primary biliary cirrhosis, *H. pylori* DNA was detected in human liver tissue samples [9]. In the gallbladder mucosa of patients with gallstones, Kawaguchi et al. found a microorganism similar to *H. pylori* in 1996 [13].

Characteristics of gallstone types in an Iranian population have not been well studied before and there are very limited studies on the demographic pattern of stone types in our country [14], so this study is one of the first studies on its kind on the epidemiology of gallstone types in Iran. As information on chemical components of the stone will help in the management and prevention of gallstones [14], in this study, we aimed to do chemical component analysis of gallstones including cholesterol, bilirubin, and calcium. Given the conflicting reports about the relationship between *H. pylori* infections and gallstone formation, this study aimed to investigate the relationship between *H. pylori* positivity in the bile specimen of Iranian patients with cholelithiasis and formation and type of stone.

## 2. Materials and Methods

This prospective study reviewed a total of 196 patients who randomly allocated for cholecystectomy due to symptomatic cholelithiasis at Shahid Beheshti Training and Research Hospital affiliated to the Yasuj University of Medical Sciences between September 2016 and May 2019. Exclusion criteria were acute cholecystitis, cholangitis, biliary and hepatic tumors, Crohn's disease, previous gastric surgery, pregnancy, patients undergoing endoscopic retrograde cholangiopancreatography (ERCP), those who had used antibiotics 4–6 weeks before cholecystectomy, and patients who did not wish to take part in the study. All methods were carried out in accordance with relevant guidelines and regulations. Written informed consent was obtained from each patient, and the study protocol was approved by the Research Ethics Committee of the Yasuj University School of Medicine (approval number: IR.YUMS.REC.1399.147).

The gallbladder tissue, bile, and stone samples excised during laparoscopic cholecystectomy were placed in 10% buffered formaldehyde solution immediately after cholecystectomy and sent to the pathology laboratory for histopathological assessment. Histological analysis was done using hematoxylin-eosin staining.

Chemical analysis of gallstone components performed is using the colorimetry method and includes examination of cholesterol, bilirubin, and calcium. This method is a technique in which the concentration of a particular substance is determined by measuring its absorption in a sample in the form of colored solution. The substance itself or the colored product made through a chemical reaction is used to produce the colored solution and is then measured with particular wavelength. Gallstone component chemical analysis with colorimetry method includes cholesterol, bilirubin, and calcium examination. The percentage of cholesterol in the stone determines the type of gallstone [15]. Gallstones are classified as cholesterol stone if the cholesterol content is >70%, pigment stone (black or brown) if the cholesterol content is <30%, and mixed stone if the cholesterol content is 30–70% [6].

In the sample extraction, gallstones were washed, dried superficially, and weighed to the nearest 0.1 g. After drying to constant weight (48 hours at 37°C), the stones were weighed again. Gallstones or parts of them were ground in a mortar. Thirty milligram of gallstone powder was transferred to 15 mL glass tubes [15].

Then, for the cholesterol analysis, 10 mL of ethanol was added, and the closed tubes were incubated at 50°C for 10 minutes to complete the dissolution of cholesterol. After standing at room temperature for another 10 minutes, the tubes were centrifuged at 2000 g during 10 minutes. In the next, the measurement of cholesterol level was done using the colorimetry method. Results from the chemical reaction are examined at 500 nm wavelength (Cobas Mira plus S Autoanalyzer, supplied by Roche). This assay is linear from 0 to 10 mmol/L [15].

For the analysis of total bilirubin, to a portion of 10 mg of gallstone in a glass tube, 200 *μ*Ι of dimethyl sulfoxide (DMSO) was added, and the mixture incubated for half an hour. Ten microliter of hydrochloric acid (HCl) (12 mol/L) was added, and incubation was continued for another half hour to dissociate calcium bilirubinate. Then, 5 mg of ethylenediaminetetraacetic acid (EDTA) was added to bind free calcium, and 100 *μ*Ι of NaOH (1.2 mol/L) was added to achieve a neutral pH. Results from the chemical reaction are examined at 546 nm wavelength (Cobas Mira plus S Autoanalyzer, supplied by Roche) [15].

For the analysis of calcium, the o-cresolphthalein complexon (CPC) method (nonenzymatic colorimetry) was used. A portion of 25 mg of gallstone powder was transferred to a 50 mL volumetric flask. Five drops of concentrated HCl was added, and after one minute, 1 mL of water was added. This mixture is used to analyze calcium. Results from the chemical reaction are examined at 577 nm wavelength (Ziest Chem Diagnostics Co, Tehran, Iran; calcium CPC) [16].

Some techniques are used to diagnose *H. pylori* infection, such as histopathological diagnosis, rapid urease test (RUT), microbiological culture, polymerase chain reaction (PCR), stool antigen test, serology, and urea breath test (UBT). Markedly, all these techniques have their own disadvantages. Experienced pathologists and quality of biopsies are two basic requirements for a proper histopathological examination. Improper biopsies, observer-related factors, *H. pylori* density and its patchy distribution, and type of stain used may cause false results. In clinical practice, the most routinely used technique is RUT. However, to obtain a sufficient sensitivity, there should be sufficient bacterial load consisting of at least 10^5^ bacteria. Bacterial culturing is regarded as a definite proof of *H. pylori* infection, although the ability to culture and the sensitivity of the test may vary between laboratories [17]. Limitations of PCR methods include the propensity for false-positive results in part due to the detection of cDNA from non-*H. pylori* organisms. This is particularly important in environmental samples that may contain previously uncultured organisms or non-*H. pylori Helicobacter* spp. False-negative results may also occur due to a low number of organisms or to the presence of inhibitors in the sample [18]. Serology is the easiest way to detect *H. pylori* infection by detecting circulating antibodies against *H. pylori*. However, it cannot differentiate between active and asymptomatic colonization and past and current *H. pylori* infection. The UBT has a greater sensitivity and specificity, but its specificity is decreased when other urease-producing bacteria are present in the human gut. It also needs more expensive and complicated equipment [17]. The stool antigen test for *H. pylori* is a rapid, easy, noninvasive, and inexpensive method for detecting *H. pylori* infection. This test showed a high sensitivity and specificity [19].

In this study, for the first time, microbiological analysis of the bile sample for *H. pylori* was done using the OnSite *H. pylori* Rapid Stool Ag Test. To validate the test on bile, the *H. pylori* Rapid Stool Ag Test on stool samples of all the patients were done with the same kit and method. The rapid antigen test method is a lateral flow chromatographic immunoassay for the qualitative detection of *H. pylori* antigen. This uses a colloidal gold conjugated monoclonal anti-*H. pylori* antibody and another monoclonal anti-*H. pylori* antibody to specifically detect *H. pylori* antigen present in the fecal or other specimens of an infected patient (CTK Biotech, Inc., San Diego, USA; Catalog Number R0192C) [20]. In this study, this test was used for the detection of *H. pylori* in the bile and stool samples.

Briefly, the stool sample gathered from the patient before the operation was sent to the laboratory from the surgery ward; the bile sample was collected in a syringe in sterile conditions and was sent to the laboratory for analysis at the time of laparoscopic cholecystectomy from the operation room. Small samples of stool were transfered to a vial with diluent vigorously agitated and after two minutes of resting the tube, dropping around two to three drops into the round window of the test cassette. (The same method was used for bile.) Reading was made after 10 minutes of incubation at room temperature, and based on the appearance of colored lines across the central window of the cassette, two lines, C (control) and T (test), indicated positive test, and only one line in C indicated negative result. A pale colored line in T was also considered positive.

Data were analyzed using IBM Statistical Package for the Social Sciences (SPSS) Statistics for Windows, version 22.0, software (IBM Corp., Armonk, NY, USA). Descriptive statistics were given as mean ± standard deviation (SD), median (maximum, minimum), number (*n*), and percentage (%). Conformity of the data to a normal distribution was assessed using the Kolmogorov–Smirnov test. Quantitative independent data were analyzed using the Mann–Whitney *U* test. Qualitative independent data were analyzed by the chi-squared test or by Fisher's test if the chi-squared test conditions were not met. Cohen's Kappa test is used to measure the degree of agreement between the two methods (rapid antigen test in bile and rapid stool antigen test for *H. pylori*). *P* < 0.05 was accepted as statistically significant.

## 3. Results

One hundred ninety-six patients were entered in the study, of whom 33 (16.9%) were men and 163 (83.1%) were women. Mean age in male and female groups were 48.6 ± 16.8 and 47.0 ± 17.4, respectively. The youngest and the oldest patients were 19-year-old and 90-year-old, respectively. There was no significant association between age and sex (*P* value = 0.51). The most common drugs used were proton pump inhibitors (PPIs) (pantoprazole, esomeprazole, and omeprazole) in 144 (73.4%), nonsteroidal anti-inflammatory drugs (NSAIDs) or pain killers in 87 (44.3%), antihypertensive drugs in 43 (21.9%), and oral contraceptives (OCPs) in 27 (13.7%).

**Figure 1:**
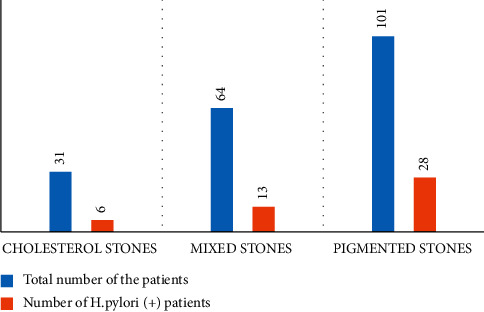
Number of the patients according to the stone types and *H. pylori* positivity.

Stone types were as follows: cholesterol stones in 31 (15.8%) patients, mixed stones in 64 (32.6%) patients, and the most common type, the pigmented stones in 101 (51.5%) patients. *H. pylori* was positive in 47 (24.0%) patients of whom 6 (12.8%), 13 (27.7%), and 28 (59.6%) had cholesterol, mixed, and pigmented stones, respectively (Figure 1). However, there was no significant association between *H. pylori* positivity and stone type (*P* value = 0.447).

Mean age in *H. pylori*-positive and *H. pylori*-negative groups was 49.8 ± 16.3 and 46.4 ± 17.5 years, respectively. There was no association between *H. pylori* positivity and age (*P*value = 0.15).

Of the 47 patients with positive result for *H. pylori*, 12 (25.5%) and 35 (74.4%) patients were male and female, respectively. There was no significant association between *H. pylori* positivity and sex (*P* value = 0.06).

Forty-seven (23.9%) of the 196 patients were positive according to the rapid antigen test, being defined as *H. pylori*-positive in bile, and 149 patients were negative according to the rapid antigen test and considered *H. pylori*-negative in bile. In the *H. pylori*-positive group, the rapid stool antigen test detected *H. pylori* antigen in stool of all the patients (sensitivity 77%; 95% confidence interval (CI): 64.50–86.85%), and 14 false-negatives (FN); and in the *H. pylori*-negative group, 135 presented negative results (specificity 100%; 95% CI: 97.30–100.00%), and no false-positives (FP), showing a substantial agreement (Kappa Index = 0.822; CI: 0.73–0.91). Considering that 47 patients of the 61 that had positive stool antigen were *H. pylori*-positive according to the rapid antigen test in bile, the positive predictive value (PPV) of the stool antigen test was 100%; and considering that 135 patients with negative stool antigen test were *H. pylori*-negative in bile, the negative predictive value (NPP) of the stool antigen test was 90.6% (95% CI: 85.89–93.85%), and the accuracy of the test was 92.86% (95% CI:88.31–96.04%), as shown in Table 1.

**Figure 2:**
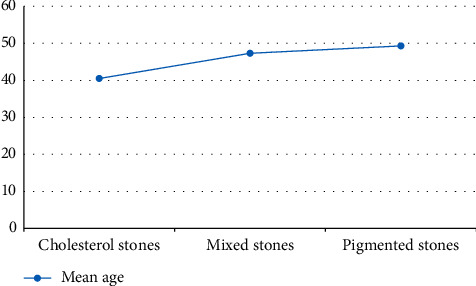
Stone types and mean age.

Mean age in different stone type groups were as follows: 40.5 ± 13.1 years in cholesterol stones, 47.3 ± 14.7 years in mixed stones, and 49.3 ± 19.4 years in pigmented stones (Figure 2). There was a significant association between stone types and mean age (*P* value = 0.047).

The numbers of male and female patients according to the type of stone are shown in Table 2. However, there was no significant association between stone type and sex (*p*-value = 0.51), and the male-to-female ratio according to stone types were 4/27, 9/55, and 20/81, respectively, which shows more men in the pigmented stone group than the cholesterol and mixed groups.

**Figure 3:**
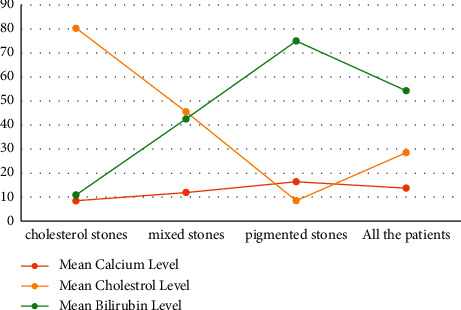
Mean calcium, cholesterol, and bilirubin level in all the patients and different types of stone.

The mean calcium level in all the patients was 13.7 ± 6.0 mmol/L. The mean calcium level in three types of stone was 8.5 ± 2.7, 11.9 ± 4.8, and 16.4 ± 6.0 mmol/L in cholesterol, mixed, and pigmented stone groups, respectively (Figure 3). Pairwise comparison of the three groups of stone types showed significant differences between them, so there was a significant association between type of stone and calcium level (*P* value < 0.05) (Table 3). The mean calcium level in *H. pylori*-positive and *H. pylori*-negative groups was 13.2 ± 5.5 and 13.8 ± 6.1 mmol/L, respectively. There was no significant association between *H. pylori* positivity and calcium level (*P* value = 0.71).

The mean cholesterol level in all the patients was 28.5 ± 2.0 mmol/L. The mean cholesterol level in three types of stone was 80.4 ± 14.1, 45.5 ± 13.2, and 8.5 ± 4.0 mmol/L in cholesterol, mixed, and pigmented stone groups, respectively (Figure 3). Pairwise comparison of the three groups of stone types according to the cholesterol level is shown in Table 3. The mean cholesterol level in *H. pylori*-positive and *H. pylori*-negative groups was 26.9 ± 28.1 and 33.6 ± 28.5 mmol/L, respectively. There was a significant association between *H. pylori* positivity and cholesterol level (*P* value = 0.023).

The mean bilirubin level in all the patients was 54.3 ± 25.9 mg/dL. The mean bilirubin level in three types of stone was 10.9 ± 12.8, 42.6 ± 13.3, and 75.0 ± 6.7 mg/dL in cholesterol, mixed, and pigmented stone groups, respectively. Pairwise comparison of the three groups of stone types showed significant differences between them, so there was a significant association between type of stone and bilirubin level (*P* value<0.05) (Figure 3, Table 3). The mean bilirubin level in *H. pylori*-positive and *H. pylori*-negative groups were 59.8 ± 26.6 and 52.6 ± 25.5 mg/dL, respectively. There was a significant association between *H. pylori* positivity and bilirubin level (*P* value = 0.026).

## 4. Discussion

The prevalence of gallstones is low in Asians. In Iran, many factors influence the prevalence of this disease. In a cross-sectional study done by Farzaneh Sheikh Ahmad et al. in Iran, the prevalence of gallstone disease in the cadavers was 6.3% (men 4.7%, women 8.6%), and there is no much difference between men and women, and this result is compatible with our results in which there was no significant association between stone type and sex [14]. As a matter of fact, our study is one of the first studies that is done in our country on the demographic pattern (age and sex) and its association to the chemistry of the stone types and presence of *H. pylori* in the bile.

In our study, we found no significant association between the stone types and sex, but there were significant associations between stone types and age, and chemical composition of the stones such as calcium, cholesterol, and bilirubin. In this study, we did not find any significant association between the presence of *H. pylori* and age, sex, stone types, and calcium level, but there were significant associations between *H. pylori* positivity and cholesterol and bilirubin levels. It means that the chemical composition of the stones could predict the presence of bacteria. This result is comparable with the result of a study done by Stewart et al. in which neither the chemical composition nor stone appearance predicted the presence of bacteria and that the seventy-three percent of pigment stones contained bacteria that produced beta-glucuronidase, slime, and phospholipase, factors that ease stone formation [21].

While the relation between infection and gallstones is still controversial and there is an argument on it, infection has been proposed as a reason, especially for the brown pigment stones. Several reports have shown the existence of Helicobacter species such as pylori, bilis, *Flexispira rappini*, and *pullorum* in the human hepatobiliary system in recent years. As a matter of fact, the cause of some hepatobiliary diseases such as chronic cholecystitis, primary sclerosing cholangitis, and gallbladder carcinoma could be the infection with species. The presence of *H. pylori* DNA in cholesterol gallstones could suggest that *H. pylori* is an instinctive part of a stone-forming gallbladder. By the way, colonization of the biliary tract by *H. pylori* could prompt the gallbladder to form stones [9, 11, 22, 23]. To add more, there are some studies with no association between *H. pylori* and hepatobiliary diseases, in contrast to the reports with positive association mentioned above. The results of our study are compatible with the results of the studies that found no correlation between *H. pylori* positivity and stone formation [24–27].

For the validation test of bile, the *H. pylori* Rapid Stool Ag Test on stool was used, and Cohen's Kappa statistical analysis was done next. Cohen suggested the Kappa result be interpreted as follows: Kappa < 0: no agreement; Kappa between 0.00 and 0.20: slight agreement; Kappa between 0.21 and 0.40: fair agreement; Kappa between 0.41 and 0.60: moderate agreement; Kappa between 0.61 and 0.80: substantial agreement; and Kappa between 0.81 and 1.00: almost perfect agreement [28]. Our results were matched to the substantial agreement between the two tests and were acceptable.

Some drugs are reported to make gallbladder stones by different pathogenetic mechanisms, such as estrogens, progesterone, oral contraceptives, clofibrate, ceftriaxone, octreotide, erythromycin, ampicillin, thiazide diuretics, somatostatin analogs, cyclosporin, dapsone, anticoagulant treatment, and narcotic and anticholinergic medications [29, 30]. In our study, PPI was the most common drug used. In the other studies, it is shown that short-term PPI therapy reduces gallbladder motility in healthy volunteers [31]. In our study, the second most common drug used was NSAIDs. Minocha et al. reported no significant difference between the number of subjects taking NSAIDs chronically and those not taking NSAIDs according to gallstones [32]. In our study, hydrochlorothiazide was used in 9 out of 43 patients (20.9%) with antihypertensive drug. Leitzmann et al. hypothesized that the use of thiazide diuretics increases the risk of symptomatic cholecystitis [33]. In our study, OCPs were used in 16.5% of the women. A meta-analysis showed a significant increase in the formation of gallbladder stones among women who were using oral contraceptives [34]. Both estrogen and progesterone have been shown to increase the risk of gallstones. Estrogen would increase cholesterol production in the liver, and progesterone would decrease gallbladder motility [35].

## 5. Conclusions

In conclusion, this study suggested that the chemical composition of the stones could predict the presence of bacteria, there is no correlation between *H. pylori* and gallstone formation, and some of the drugs could be predisposing factors for gallstones. This work provides an objective basis for further research into gallbladder stone formation; meanwhile, it has great significance in the treatment and prevention of gallbladder stones.

## Figures and Tables

**Table 1:** Performance of the rapid antigen test in bile using the rapid stool antigen test to define *H. pylori*-positive and *H. pylori*-negative groups.

Group	Method	TP	TN	FP	FN	Kappa Index	Sensitivity (%)	Specificity (%)	PPV (%)	NPV (%)	Accuracy (%)
196	Rapid antigen test in bile	47	135	0	14	0.822	77	100	77	90.6	92.86

**Table 2:** Number of male and female patients according to the type of stone.

	Cholesterol stones	Mixed stones	Pigmented stones	Total
Male	4	9	20	33
Female	27	55	81	163
Total	31	64	101	196

**Table 3:** Pairwise comparison of the three groups of stone types and their *p*-values for calcium, cholesterol, and bilirubin.

Sample1-Sample2	Calcium *P* value	Cholesterol *P* value	Bilirubin *P* value
Cholesterol-mix	0.007	0.001	0.001
Cholesterol-pigmented	*P* ≤ 0.001	*P* ≤ 0.001	*P* ≤ 0.001
Mixed-pigmented	*P* ≤ 0.001	*P* ≤ 0.001	*P* ≤ 0.001

## Data Availability

The data sets used and/or analyzed during the current study are available from the corresponding author on reasonable request.

## Abbreviations

DMSO: Dimethyl sulfoxide
ERCP: Endoscopic retrograde cholangiopancreatography
*E. coli*: *Escherichia coli*
EDTA: Ethylenediaminetetraacetic acid
FN: False-negative
FP: False-positive
*H. pylori*: *Helicobacter pylori*
HCl: Hydrochloric acid
MALToma: Lymphoma of gastric mucosa-related lymphoid tissue
NPV: Negative predictive value
NSAIDs: Nonsteroidal anti-inflammatory drugs
*n*: Number
CPC: o-Cresolphthalein complexon
OCPs: Oral contraceptives
PCR: Polymerase chain reaction
PPV: Positive predictive value
PPIs: Proton pump inhibitors
RUT: Rapid urease test
SD: Standard deviation
SPSS: Statistical Package for the Social Sciences
TN: True negative
TP: True positive
UBT: Urea breath test
YUMS: Yasuj University School of Medicine.
